# Respiration Facilitates Behavior During Multisensory Integration

**DOI:** 10.1111/psyp.70145

**Published:** 2025-09-25

**Authors:** Martina Saltafossi, Andrea Zaccaro, Daniel S. Kluger, Mauro Gianni Perrucci, Francesca Ferri, Marcello Costantini

**Affiliations:** ^1^ Institute for Biomagnetism and Biosignal Analysis University of Münster Münster Germany; ^2^ Otto Creutzfeldt Center for Cognitive and Behavioral Neuroscience University of Münster Münster Germany; ^3^ Department of Psychology “G. d'Annunzio” University of Chieti‐Pescara Chieti Italy; ^4^ Department of Neuroscience, Imaging and Clinical Sciences “G. d'Annunzio” University of Chieti‐Pescara Chieti Italy; ^5^ Institute for Advanced Biomedical Technologies (ITAB) “G. d'Annunzio” University of Chieti‐Pescara Chieti Italy

**Keywords:** brain–body interactions, interoception, multisensory integration, perception, respiration

## Abstract

The brain processes information from the external environment alongside signals generated by the body. Among bodily rhythms, respiration emerges as a key modulator of sensory processing. Multisensory integration, the non‐linear combination of information from multiple senses to reduce environmental uncertainty, may be influenced by respiratory dynamics. This study investigated how respiration modulates reaction times and multisensory integration in a simple detection task. Forty healthy participants were presented with unimodal (Auditory, Visual, Tactile) and bimodal (Audio‐Tactile, Audio‐Visual, Visuo‐Tactile) stimuli while their respiratory activity was recorded. Results revealed that reaction times systematically varied with respiration, with faster responses during peak inspiration and early expiration but slower responses during the expiration‐to‐inspiration transition. Applying the race model inequality approach to quantify multisensory integration, we found that Audio‐Tactile and Audio‐Visual stimuli exhibited the highest integration during the expiration‐to‐inspiration phase. These findings conceivably reflect respiration phase‐locked changes in cortical excitability which in turn orchestrates multisensory integration. Interestingly, participants also tended to adapt their respiratory cycles, aligning response onsets preferentially with early expiration. This suggests that, rather than a mere bottom‐up mechanism, respiration is actively adjusted to maximize the signal‐to‐noise balance between interoceptive and exteroceptive signals.

## IMPACT STATEMENT

Our findings deepen growing interest in how exteroception and interoception interact. We show that respiration modulates speeded responses and multisensory integration, with peak effects during transition phases, suggesting active coordination between bodily rhythms and sensory processing to optimize perception.

## 1 Introduction

The brain dynamically interacts with the external world, processing information from the five senses and guiding behavioral responses. Current research attempts to recast neural implementation of cognition to include the modulatory effects of peripheral bodily signals, that is, interoception (Azzalini et al. 2019; Critchley and Garfinkel 2018; Kluger, Allen, and Gross 2024). Human respiration represents a unique interoceptive system because, besides being a simple reflex, it can also be under volitional control (Herrero et al. 2018; Maric et al. 2020; McKay et al. 2003). In addition, breathing patterns are continuously adjusted to match not only metabolic demands, but also vocal behaviors (e.g., speaking, crying, laughing, singing) as well as vital functions (e.g., swallowing and suckling) (Abbasi et al. 2023; Feldman et al. 2013; McKay et al. 2003). Recent animal and human studies revealed a profound intertwining between the respiratory cycle and spontaneous brain activity, extending beyond homeostatic and allostatic needs (Brændholt et al. 2023). Through multiple, and not necessarily independent, olfactory, somatosensory, interoceptive, and chemoreceptive pathways and mechanisms (Allen et al. 2023), respiration drives changes in the amplitude of brain oscillations (Fontanini and Bower 2006; Ito et al. 2014; Karjalainen et al. 2023; Kluger and Gross 2020, 2021; Kluger et al. 2021; Tort et al. 2018), shifts of cortical and cortico‐spinal excitability (Engelen et al. 2024; Kluger et al. 2023, 2025), and modulations of neuronal spiking in cortical and subcortical structures (De Falco et al. 2024; Ito et al. 2014; Yanovsky et al. 2014).

As a functional consequence of this respiratory entrainment of neural dynamics, cycle‐to‐cycle coupling of breathing and behavior has been repeatedly found (Heck et al. 2022; Johannknecht and Kayser 2022; Parviainen et al. 2022; Varga and Heck 2017). For instance, inspiration has been shown to improve recognition of fearful faces (Zelano et al. 2016), memory of odors (Arshamian et al. 2018), accuracy in visuospatial (Kluger et al. 2021; Perl et al. 2019) and perceptual decision‐making tasks (Brændholt et al. 2024), as well as to reduce reaction times (RTs) to visual (Flexman et al. 1974) and auditory stimuli (Gallego et al. 1991). A similar phase dependency has been revealed for interoceptive attention optimization (Zaccaro et al. 2022, 2024), startle responses to auditory inputs (Münch et al. 2019), conscious tactile perception (Grund et al. 2022), conditioned learning (Waselius et al. 2019, 2022), and action execution (Park et al. 2020, 2022) all being boosted by expiration. From a predictive processing perspective, which posits that perception results from minimization of prediction errors (PEs) (i.e., the discrepancy between priors and sensory input) (Friston 2009), such covariation between respiration and behavior has been interpreted as an *active sensing* mechanism (Schroeder et al. 2010). This hypothesis rests on animal studies demonstrating that rodents' sensory‐motor routines (e.g., orofacial movements like sniffing and whisking) are gated to the respiratory rhythm (Corcoran et al. 2018; Kurnikova et al. 2017; Wachowiak 2011). Similarly, human participants spontaneously adapt their respiratory cycle to the onset of stimuli and responses, preferentially matching inspiration or expiration, likely depending on task difficulty and sensory modality (Grund et al. 2022; Harting et al. 2024; Johannknecht and Kayser 2022; Perl et al. 2019; Zelano et al. 2016). By aligning events with respiration, the brain might fine‐tune neural processes underlying adaptive behavior (Allen et al. 2023; Brændholt et al. 2023; Park et al. 2020).

Overall, the research examined so far highlights the crucial role of respiration in regulating information gating and interaction with the environment, by acting as a “clock mechanism” (Corcoran et al. 2018; Kluger et al. 2021; Kurnikova et al. 2017). Through its rhythmic nature, respiration creates recurring windows of heightened and diminished neural excitability, which are thought to optimize the timing of sensory sampling and integration of interoceptive and exteroceptive information, ultimately structuring perception and action (Chalas et al. 2025). However, studies on respiratory modulations of perception have largely investigated one single sensory modality at a time. Hence, it remains unknown whether and how respiration modulates perception of everyday multisensory stimuli. Here, each conscious percept stems from the continuous combination and integration of multiple sensory inputs from the external world, which results in enhanced neural and behavioral outputs (Laurienti et al. 2005; Sperdin et al. 2010; Stein 1998; Stein et al. 2020). This facilitation provided by the synthesis among multiple sources of information is defined as multisensory integration (MI) and is experimentally assessed by comparing responses to cross‐modal stimuli with those of the corresponding unisensory stimuli (i.e., effectiveness) (Meredith and Stein 1983; Rowland et al. 2007; Stein and Stanford 2008).

We recently demonstrated a significant interplay between exteroceptive (multisensory) and interoceptive processes by showing that the cardiac cycle modulates multisensory integration, likely through competition‐like suppression of tactile stimuli during systole (Saltafossi et al. 2023). Here, we extended this investigation to explore the coupling between respiration and multisensory perception. We subjected participants to a simple detection task involving three sensory modalities (i.e., hearing, touch, and vision), presented alone (unimodal) or in combination (bimodal). We first hypothesized that speeded responses to unimodal and bimodal inputs would vary systematically across phases of spontaneous respiration. Second, after assessing super‐additive interactions during bimodal stimulations, we tested whether the magnitude of multisensory integration was modulated across four respiratory phase bins, segmenting the cycle into transition (inspiration‐to‐expiration and expiration‐to‐inspiration) and nontransition (inspiration and expiration) phases. On the behavioral level, we examined the alignment of response onsets to respiration phase in keeping with previous reports of sensory‐motor coupling (see above).

## 2 Materials and Methods

### 2.1 Participants

Forty‐one participants (29 female; 3 left‐handed; mean age ± SD = 24.88 ± 2.90 years) took part in the study, recruited from the “Gabriele d'Annunzio” University of Chieti‐Pescara and the wider community. All participants had normal or corrected‐to‐normal vision. Exclusion criteria for participating were self‐reported history of hearing loss and either mental, cardiovascular, or neurological disorders. Before the experiment, participants gave written informed consent. Ethical approval from the local ethics board was obtained (Institutional Review Board of Department of Psychological, Health and Territorial Sciences, “Gabriele d'Annunzio” University of Chieti‐Pescara, Protocol Number 23013). The experiment was conducted following the Declaration of Helsinki.

Analyses were performed on 40 participants after excluding one participant due to excessive missed responses (> 50%). Block 1 was excluded from two participants due to failures in data recordings. A total of 28,320 trials were analyzed.

### 2.2 Experimental Setup

**FIGURE 1:**
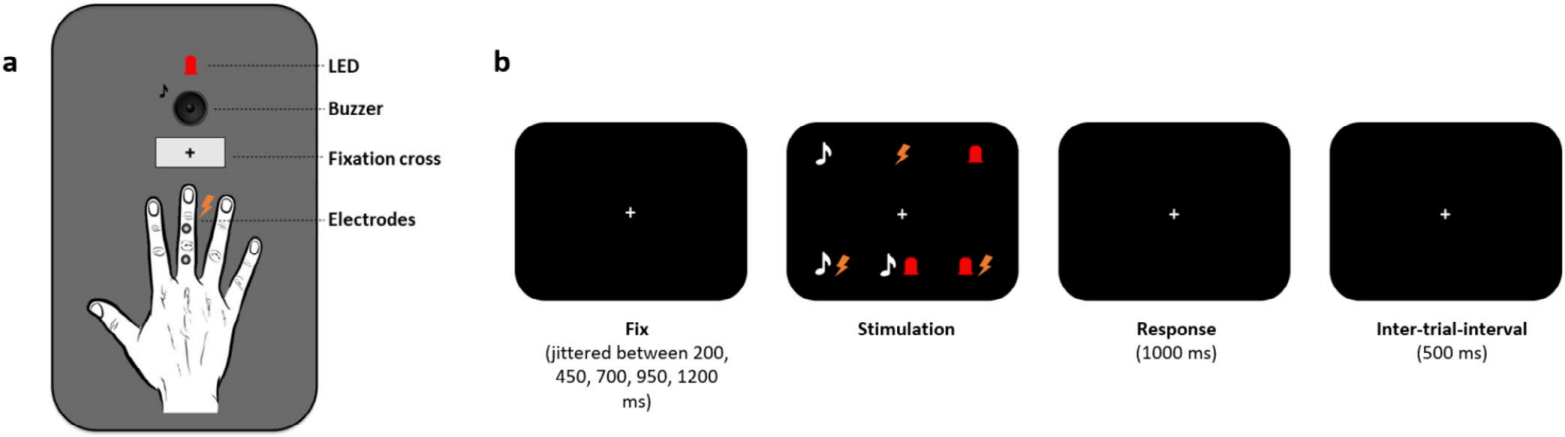
Experimental setup. (a) Stimulus delivery apparatus: The in‐house box presented electric pulses as tactile stimuli, brief flashes as visual stimuli (LED), and auditory stimuli via a buzzer, in close proximity. (b) Timeline of trials: After a period with a variable duration, a stimulation occurred (either unimodal or bimodal). Participants had to respond within 1000 ms by pressing a pedal. The response window was then followed by an inter‐trial interval of 500 ms.

The stimulus delivery apparatus was identical to that described in (Saltafossi et al. 2023) (Figure 1a). The stimulation included three unimodal stimuli—Auditory (A), Tactile (T), and Visual (V)—as well as their bimodal combinations: Audio‐Tactile (AT), Audio‐Visual (AV), and Visuo‐Tactile (VT). For a detailed explanation of the stimulus types and thresholding procedure, readers are referred to Supporting Information S1 and Saltafossi et al. (2023). Respiratory activity was recorded with a BIOPAC MP160 (BIOPAC System Inc., Goleta, CA, USA) (Low‐pass filter: 35 Hz; high‐pass filter: 0.05 Hz; notch filter: 50 Hz; sampling rate: 2000 Hz) using AcqKnowledge software (version 5.0.5, BIOPAC System Inc., Goleta, CA, USA). A respiration belt with a transducer was placed around the participants' chest.

The experiment design mirrored that of Saltafossi et al. (2023) but stimuli were not triggered by cardiac or respiratory phases. In a simple detection task, participants fixated on a central cross and responded quickly to stimuli. After a variable delay, randomly selected from fixed durations of 200, 450, 700, 950, or 1200 ms, unimodal (A, T, V) or bimodal (AT, AV, VT) stimuli were presented. Participants pressed a pedal with their right foot upon perceiving a stimulus (either unimodal or bimodal), with a maximum response time of 1000 ms. Each trial had a fixed 500 ms inter‐trial interval (Figure 1b). A total of 720 trials, presented in pseudorandom order, were divided into three blocks of 240 stimuli, equally split by type. A brief training session familiarized participants with the task. Reaction times were recorded using a pedal board connected to the Trigger Station (BRAINTRENDS LTD 2010, Rome, Italy). Breaks were allowed between blocks to prevent fatigue and maintain focus.

### 2.3 Redundant Signal Effect Analysis

The redundant signal effect (RSE (Hershenson 1962; Todd 1912)) occurs when individuals are asked to make fast responses in a simple detection task. This effect reflects the relative gain in RTs, observed when stimuli are presented simultaneously in multiple sensory modalities (i.e., redundant stimuli), as opposed to a single modality (Diederich and Colonius 2004; Hershenson 1962; Swinkels et al. 2021; Todd 1912). We adjusted reaction time data as follows. First, responses faster than 120 ms were classified as “fast guesses” and were removed from the analysis (Couth et al. 2018). Next, for each stimulus type and participant, we trimmed all RTs falling outside 2 SD from the mean (5.09% of data was rejected from raw RTs). Finally, we assessed the normality of data distributions using Lilliefors tests for each stimulus type, implemented in MATLAB (R2023a, MathWorks Inc., Natick, MA, USA). To quantify the redundant signal effect, we computed Friedman tests for each of the modality triplets (A/T/AT, A/V/AV, V/T/VT) using the MATLAB *friedman* function. Where necessary, post hoc analyses were conducted by comparing each bimodal condition with its respective unimodal condition and applying Tukey–Kramer multiple comparisons correction. These comparisons were conducted using MATLAB's *multcompare* function.

### 2.4 Race Model Inequality Analysis

Several models have been proposed to explain RSE, including the so‐called *race models*. In race models, the two components of a bimodal stimulation are processed in separate sensory channels, and the faster channel determines processing time (Colonius et al. 2017; Gondan and Minakata 2016). Therefore, these models imply that RSE is a mere consequence of statistical facilitation or probability summation (Raab 1962), overlooking super additive enhancements from multisensory integration (Colonius and Diederich 2017). To distinguish between separated processing (race model) and integrated processing (multisensory integration), Miller (1982) derived *race model inequality* (RMI), which has become an important testing tool for redundancy gains analysis (Gondan and Minakata 2016; Miller 1982, 1986). RMI states that the cumulative RT distribution for the redundant stimuli never exceeds the sum of RT distributions for the unimodal stimuli, while rejection or violation of the inequality reflects multisensory integration (Gondan and Minakata 2016; Gondan 2010; Miller 1982). We tested for such RMI violations by following the procedure reported in Saltafossi et al. (2023), based on Mahoney and Verghese (2019). We organized raw RTs into 21 progressively increasing time bins. This involved determining a specific RT range for each participant by subtracting their slowest RT from the fastest RT. Subsequently, we incrementally added 5% of this range to each time bin. The cumulative distribution frequency (CDF) was then constructed by summing the total probabilities across the quantized bins, resulting in 21 time bins (0%, 0% + 5%, 0% + 5% + 10%, etc.) for each of the three multisensory pairs (AT, AV, VT). We computed the independent version of the race model (Stevenson et al. 2014) using the following formula:

$$\mathrm{CDF}\left({\mathrm{Uni}}_{x}\right)+\mathrm{CDF}\left({\mathrm{Uni}}_{y}\right)-\mathrm{CDF}\left({\mathrm{Uni}}_{x}\right)\times \mathrm{CDF}\left({\mathrm{Uni}}_{y}\right)$$

Individual RMI values were generated for each time bin by subtracting the predicted CDF (i.e., independent race model) from the actual CDF representing multisensory conditions. Violations of RMI occur when probability difference values (i.e., actual CDF—predicted CDF) are positive. However, to probe whether there was a statistically significant violation while controlling for Type I error (Kiesel et al. 2007), a series of permutation tests (*n* = 10,001) were run over the violated portion (i.e., positive values) of the group‐averaged difference wave, using MATLAB *rmiperm* function (Gondan 2010). Results provide a *T*_max_ value, 95% criterion *T*_critic_ value, and *p*‐value to determine whether multisensory integration took place across the study sample (Mahoney and Verghese 2019, 2020).

### 2.5 Statistical Analysis Relating Respiration and Reaction Times

Respiratory data were processed using custom MATLAB scripts. Respiration phase angles were extracted according to a well‐validated procedure (Kluger et al. 2021, 2023, 2025). Specifically, points of peak inspiration (peaks) and expiration (troughs) were identified within the normalized (z‐scored) respiratory signal using MATLAB's *findpeaks* function with parameter adjustments. Then, respiration cycles were centered around peak inspiration (phase 0) through double interpolation: phase angles were linearly interpolated from trough to peak (−π to 0) and vice versa, from peak to trough (0 to π).

To relate respiration to behavioral data, we carried out bin‐wise analyses on RTs collapsed across conditions (regardless of the stimulus type) and on condition‐dependent RTs (i.e., unimodal and bimodal). Thus, we first partitioned the respiratory cycle into 60 equidistant and overlapping phase bins. Moving along the cycle in increments of Δω = π/30, we aggregated trimmed RTs according to the stimulus onset phase computed at a respiration angle of ω ± π/10 (Kluger et al. 2021). Respiration phase (bin)‐dependent averaged RTs were obtained for each participant. Finally, RTs were *z*‐scored and averaged across participants, resulting in the grand average phase‐dependent RTs at the population level. Likewise, for each condition (unimodal and bimodal) we derived the grand average RTs.

We employed a linear mixed effect model (LMEM) to investigate whether respiration affects speeded responses through the MATLAB *fitlme* function. A first (base) model predicted RTs as a combination of the fixed effect of stimulus type (i.e., unimodal and bimodal) and the random intercept for participants. We used the following formula defined according to Wilkinson notation:

$$\mathrm{RT}\;\text{trimmed}\sim 1+\text{stimulus type}+\left(1|\text{participant}\right)$$

An alternative LMEM modeled the same variables adding the fixed effect of respiration angle (with separate sine and cosine contributions) as follows:

$$\mathrm{RT}\;\text{trimmed}\sim 1+\text{sine}+\text{cosine}+\text{stimulus type}+\left(1|\text{participant}\right)$$

The MATLAB *compare* function was employed to contrast the two LMEMs, thereby assessing the modulatory effect of respiration on RTs through a theoretical likelihood ratio test (LRT). To further evaluate the significance of this respiration modulatory effect, the LMEM beta weights for the sine and cosine factors were combined into a respiratory phase vector norm, defined as:

$$\nu =\sqrt{\beta_{1}^{2}+\beta_{2}^{2}}$$

This empirical combination of sine and cosine components was tested against a null distribution generated from randomized individual RTs. Specifically, the LMEM was recalculated 1000 times, shuffling each participant's RTs across the 60 respiratory phase bins. For each iteration, the resulting beta weights for sine and cosine were combined as described above, producing a distribution of “null vector norms”. The significance of the observed empirical vector norm was determined by computing its percentile rank within the null distribution's density function.

To corroborate the LMEM findings and examine the directionality of the effect, the distributions of both overall RTs and condition‐dependent RTs across the respiratory cycle were tested for uniformity using the Rayleigh test provided by the *circstat* toolbox (Berens 2009) running on MATLAB. Only for the condition‐dependent RTs, a Watson‐Williams (WW) test was performed, contrasting their circular means to check whether RTs following unimodal and bimodal trials were differentially modulated by respiration. To test for significant respiration‐related changes in RTs (e.g., lowering and increasing), z‐scored RTs were first transformed into t‐values and then subjected to two‐tailed t‐tests to determine whether the observed changes in the data are unlikely to occur by random chance. Significance levels were adjusted for false discovery rate (FDR) using the MATLAB *mafdr* function.

### 2.6 Statistical Analysis Relating Respiration and Multisensory Integration

To investigate whether respiration played a role in shaping multisensory integration, RMI analyses were performed on 4 respiration phases, including both transition and non‐transition phases. Transition phases, that is, expiration‐to‐inspiration (ex2in) and inspiration‐to‐expiration (in2ex), were defined as ¾π to −¾π and −π/4 to π/4, respectively, while non‐transition phases, that is, ongoing inspiration and expiration, were defined as −¾π to −π/4 and π/4 to ¾π (Kluger et al. 2025). For bimodal conditions where the rejection of the inequality was observed, trials were clustered into the four phases according to their stimulus onset phase. As RMI analysis requires a comparable number of observations between conditions with a minimum of 20 (Kiesel et al. 2007), trials were down‐sampled for each participant using the MATLAB *sampleDown* function provided by the *RSE‐box* toolbox (Otto 2019). Additionally, participants with a low number of trials (< 20) for each stimulus type and respiratory phase were excluded from the analysis (6 participants for AT‐ex2in, 6 for AT‐inspiration, 4 for AT‐expiration, 2 for AT‐in2ex, 6 for AV‐ex2in, 5 for AV‐inspiration, 3 for AV‐in2ex, and 4 for AV‐expiration). In this approach, the down‐sampled raw reaction times were subjected to RMI analyses, as previously described. This process produced group‐averaged RMI waveforms for each of the four phases across all bimodal conditions.

Aiming to quantify the difference in multisensory integration between respiratory phases, we calculated individual area‐under‐the‐curve (AUC) values of RMI wave (actual CDF—predicted CDF). AUC served to determine the magnitude of multisensory integration (MMSI) as described in (Basharat et al. 2019; Mahoney and Verghese 2019; Saltafossi et al. 2023). Therefore, RMI values from the first time bin (0%) were summed with RMI values obtained from the second time bin (5%) and divided by two (AUC 1 = (0% + 5%)/2). This process was repeated for the subsequent time bins until the last violated (positive) time bin was reached. Finally, to analyze the effect of the respiratory phase on the magnitude of multisensory integration, we set up a model comparison of two LMEMs for each bimodal condition. The first (base) model, including only the effect of the AUC window and the random effect of participants, was defined (in Wilkinson notation) as:

$$\text{MMSI}\sim 1+\mathrm{AUC}+\left(1|\text{participant}\right)$$

We entered as many AUCs as long as at least one phase still had a positive RMI value (from AUC 1 to AUC 11). The second model added to the previous one the categorical information of the respiratory phase (ex2in, inspiration, in2ex, expiration):

$$\text{MMSI}\sim 1+\text{phase}+\mathrm{AUC}+\left(1|\text{participant}\right)$$

To substantiate whether the addition of the respiratory information improved the model fit, we contrasted LMEMs with and without the respiratory phase predictor using theoretical LRT provided by the MATLAB *compare* function. ANOVAs were performed to determine if the coefficient estimates were significantly different from zero. Last, KW tests and multiple comparisons corrected post hocs were run, using MATLAB *kruskalwallis* and *multcompare* (with Tukey–Kramer critical value type) functions, respectively, to show which phase was associated with greater MMSI.

### 2.7 Statistical Analysis of the Alignment Between Respiration and Response Onsets

To investigate whether participants temporally adjusted their respiration rhythm to response onsets, we first extracted respiration phases of the response onsets for all trials (regardless of the stimulus type) and then separately for unimodal and bimodal trials. Circular statistics (Rayleigh tests, *circstat* toolbox (Berens 2009)) were applied to test the hypothesis that, across participants, response onsets‐averaged respiratory phase values were not distributed uniformly across the cycle.

## 3 Results

### 3.1 Redundant Signal Effect

We recorded RTs during a simple detection task in which supra‐threshold unimodal (A, T, V) and bimodal (AT, AV, VT) stimuli were presented. Across participants, we observed an average hit rate above 85% for all stimulus types, which was in line with previous literature positing ceiling performance as an assumption for the RMI analysis (Otto 2019).

**FIGURE 2:**
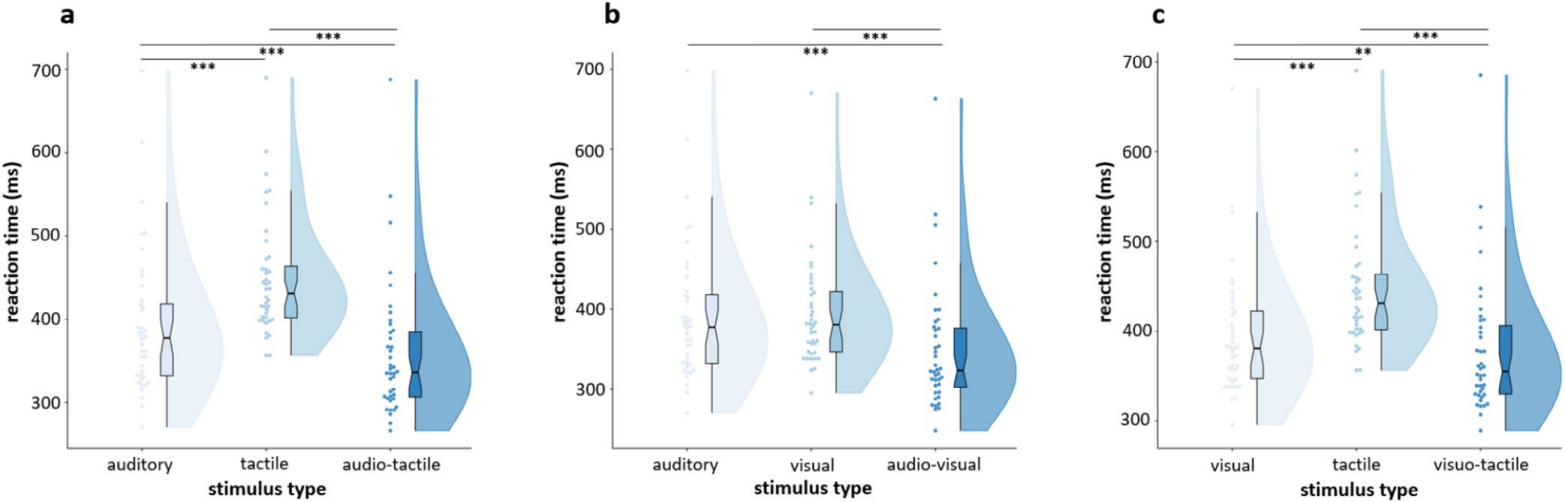
Redundant signal effect. Raincloud plots showing the behavioral facilitation related to bimodal stimulations (RSE) for each modality triplet A/T/AT (a), A/V/AV (b), and V/T/VT (c). Lines with three and two asterisks represent *p* < 0.001 and *p* < 0.01, respectively.

Since each stimulus type's data (RTs) did not follow the normal distribution (Lilliefors tests; A: *p* = 0.001; T: *p* = 0.002; V: *p* = 0.005; AT: *p* = 0.006; AV: *p* = 0.005; VT: *p* = 0.002), we investigated whether RSE was observed in each cross‐modal combination using Friedman and Tukey–Kramer post hoc tests (full results tables are reported in the Table S1). For A/T/AT F‐test, stimulus type affected RTs (χ^2^(2, 78) = 72.80, *p* < 0.001, W = 0.91), and responses to AT stimuli (Mdn = 336.40 ms) were faster than those to unimodal stimuli (vs. A (Mdn = 377.76 ms, Mdn_diff_ = −41.36 ms), *p* < 0.001; vs. T (Mdn = 431.22 ms, Mdn_diff_ = −94.82 ms), *p* < 0.001). Also, unimodal A RTs were significantly faster compared to T RTs (Mdn_diff_ = −53.46 ms, *p* < 0.001) (Figure 2a). Likewise, A/V/AV F‐test revealed a significant association of stimulus type and RTs (χ^2^(2, 78) = 60.80, *p* < 0.001, W = 0.76). Tukey–Kramer post hoc confirmed RSE, with AV RTs (Mdn = 323.55 ms) being faster than both A and V RTs (vs. A (Mdn_diff_ = −54.21 ms), *p* < 0.001; vs. V (Mdn = 380.85 ms, Mdn_diff_ = −57.30 ms), *p* < 0.001) (Figure 2b). Last, for V/T/VT F‐test, the effect of stimulus type was significant (χ^2^(2, 78) = 61.85, *p* < 0.001, W = 0.73), with Tukey–Kramer post hoc showing faster RTs to bimodal (Mdn = 355.18 ms) compared to unimodal stimuli (vs. T (Mdn_diff_ = −76.04 ms), *p* < 0.001; vs. V (Mdn_diff_ = −25.67 ms), *p* = 0.003). Moreover, responses to V stimuli were faster than responses to T stimuli (Mdn_diff_ = −50.37 ms, *p* < 0.001) (Figure 2c). In summary, bimodal stimulations lead to faster responses compared to unimodal stimulations. This finding prompted further analysis to investigate whether the observed response speed enhancement is attributable to multisensory integration.

### 3.2 Race Model Inequality and Multisensory Integration

**FIGURE 3:**
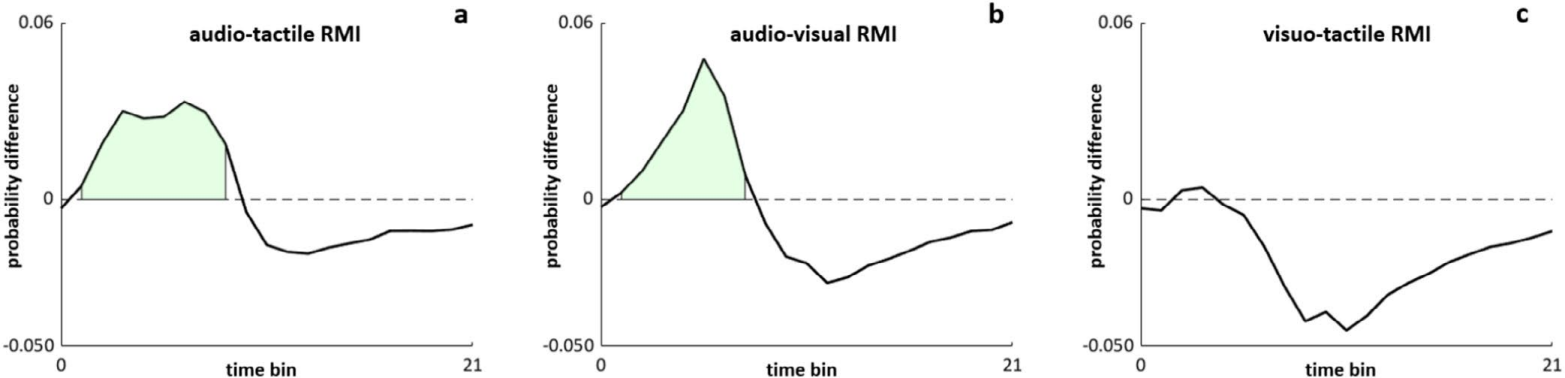
Race model inequality and multisensory integration. Group‐averaged probability CDFs difference (actual—predicted) across all time bins (from 0 to 21) are depicted for each bimodal condition. Light green areas represent the violated portion of the waveform (RMI), indicative of multisensory integration (a, b).

To test for the presence of multisensory integration, rather than only RSE, we employed Gondan's permutation tests (Gondan 2010), which iteratively compare CDFs from actual data with CDFs from the race model. AT RMI was violated within time bins ranging from the 2nd to the 8th (Tmax = 4.92, Tcritic = 2.20, p < 0.001) (Figure 3a). Similarly, AV RMI was violated over time bins 2nd to 7th (Tmax = 6.42, Tcritic = 2.08, p < 0.001) (Figure 3b). In contrast, VT did not show multisensory integration, as permutation tests over the 3rd and 4th time bins returned rejection of the alternative hypothesis (p > 0.05) (Figure 3c). While these results are confirmatory rather than novel, they pose a prerequisite for investigating respiration‐related changes in multisensory integration.

### 3.3 Respiratory Modulation of Reaction Times

We assessed whether participants' performance systematically varied over the respiratory cycle. We first demonstrated that incorporating respiratory phase sine and cosine terms into the full model, along with stimulus type information, significantly improved the fit compared to the base model (LRT‐stat χ^2^(2) = 133.16, *p* < 0.001) (all models stats are reported in the Tables S2–S3). Furthermore, the empirical vector norm, derived from combining the LMEM beta weights for sine and cosine, exceeded all null vector norms derived from 1000 iterations of randomized RTs across the 60 bins (*p* < 0.001) (see Figure S1).

**FIGURE 4:**
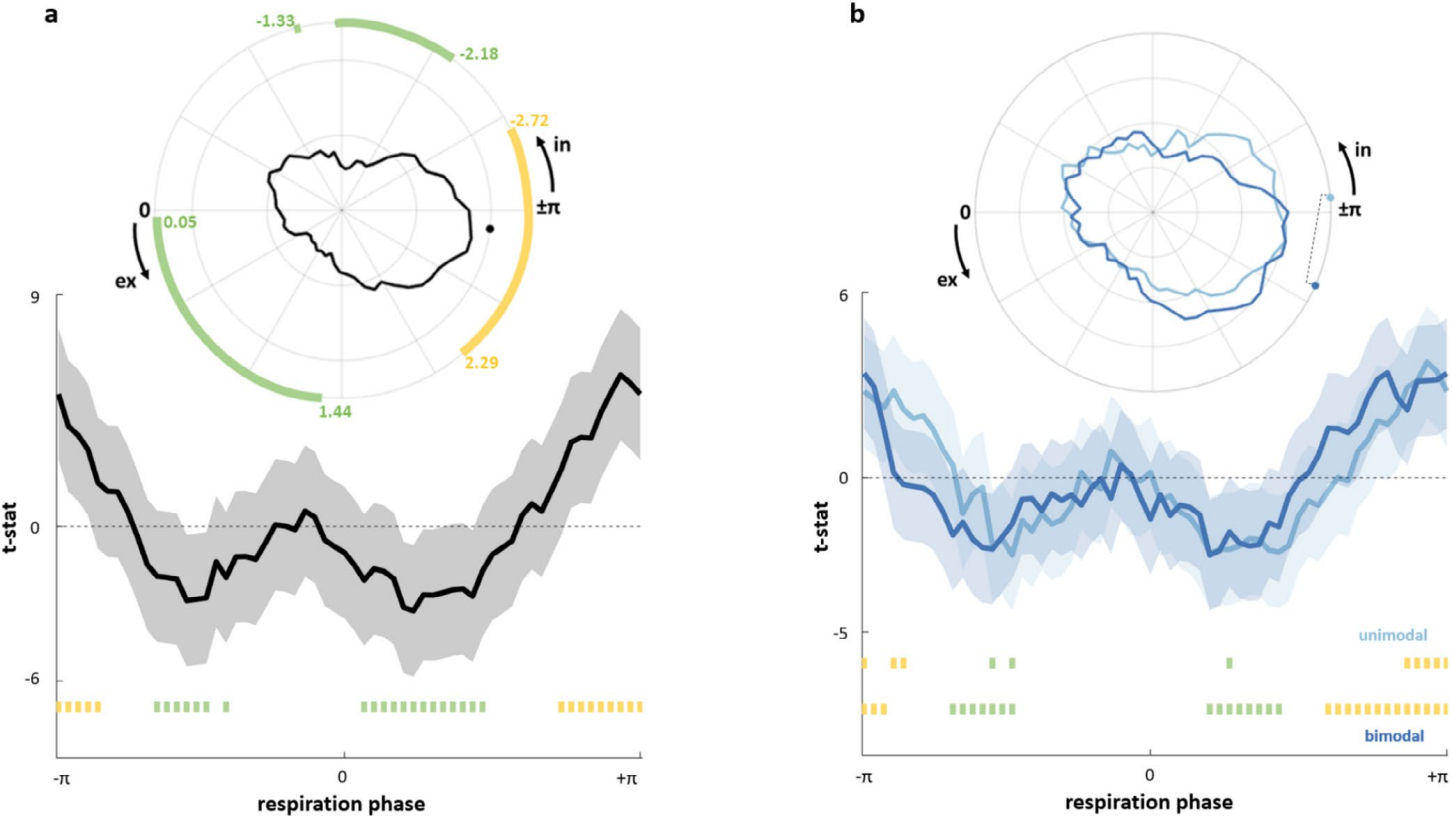
Respiratory modulation of reaction times. (a) Polar visualization shows overall RTs across the respiration cycle. A Solid black line illustrates respiration phase‐dependent RTs, black dot indicates the circular mean. Slower RTs occur in the ex2in phase (yellow markings *p*_FDR_ < 0.05), while faster RTs align with peak inspiration and early expiration (green markings *p*_FDR_ < 0.05). The 2D line graph displays t‐stat values across respiration, with shaded areas for standard deviation and green/yellow squares for significant RT changes. (b) RTs‐respiration phase comparison for unimodal (light blue) and bimodal trials (blue). In the polar plot, color‐matched dots mark circular means, connected by a dashed line for a significant Watson‐Williams test (*p* < 0.05). The 2D graph shows *t*‐stat values, with color‐coded shaded areas for standard deviation. Green and yellow squares indicate significant RT changes for each stimulation type.

Then, two separated respiratory phase bin‐wise analyses resulted in RT group distributions across the respiratory cycle, for trials pooled together (regardless of the stimulus type) and for trials separated based on the stimulus type (unimodal vs. bimodal). Through circular statistics, we confirmed the hypothesis that pooled RTs varied with respiration, regardless of the stimulus type. This was substantiated by the non‐uniform distribution of RTs (*Z*_Rayleigh_ = 4.63, *p* = 0.009), marked by a general increase towards the ex2in phase (*V*_mean_ = 3.02, 95% CI [3.70 2.34]) (Figure 4a). Importantly, respiration‐related changes in RTs were consistent across our sample: 32 participants out of 40 presented this effect (*p* < 0.05). Examining the time course of this modulation revealed slower responses within the ex2in transition (corresponding to bins' angles: −3.14 to −2.72, 2.29 to 3.14 rad, *p*_FDR_ < 0.05), while faster responses were found roughly around peak inspiration and during early expiration (corresponding to bins' angles: −2.18 to −1.44, −1.33, and 0.05 to 1.44 rad respectively, *p*_FDR_ < 0.05) (Figure 4a) (see Supplementary for *t*‐values and relative FDR adjusted *p*‐values, Table S4).

In the second circular bin‐wise analysis, both responses to unimodal and bimodal stimuli exhibited non‐uniform distributions across the respiratory cycle (unimodal *Z*_Rayleigh_ = 3.26, *p* = 0.036; bimodal *Z*_Rayleigh_ = 3.15, *p* = 0.040), but the circular means of unimodal (*V*_mean_ = −3.06, 95% CI [−2.20 to 3.92]) and bimodal stimuli (*V*_mean_ = 2.72, 95% CI [3.60 to 1.84]) differed significantly from each other (WW = 10.03, *p* = 0.002) (Figure 4b). A significant increase of RTs took place within the ex2in transition for both unimodal (bins' angles: −3.14, −2.82 to −2.72, 2.72 to 3.14 rad, *p*_FDR_ < 0.05) and bimodal trials (bins' angles: −3.14 to −2.93, 1.86 to 3.14 rad, *p*_FDR_ < 0.05), while lower RTs were mainly located towards peak inspiration and early expiration, again for both unimodal (bins' angles: −1.76, −1.54, 0.80 rad, *p*_FDR_ < 0.05) and bimodal trials (bins' angles: −2.18 to −1.54, 0.59 to 1.33 rad, *p*_FDR_ < 0.05) (Figure 4b) (see Supplementary for *t*‐values and relative FDR adjusted *p*‐values, Table S4). Respiratory modulations of RTs were observed in 34 participants out of 40, for both unimodal and bimodal stimuli (*p* < 0.05).

### 3.4 Respiratory Modulation of Multisensory Integration

Since the previous analysis had established a meaningful modulation of reaction times as a function of the respiration phase, we next addressed the question of whether respiratory phases would also modulate the magnitude of multisensory integration. To address potential changes in multisensory integration due to respiration, we set up two LMEMs for the bimodal conditions that demonstrated multisensory integration, specifically AT and AV stimuli, as detailed above. The base model, featuring only the AUC predictor, was contrasted with a second LMEM, which included the respiratory phase information.

**FIGURE 5:**
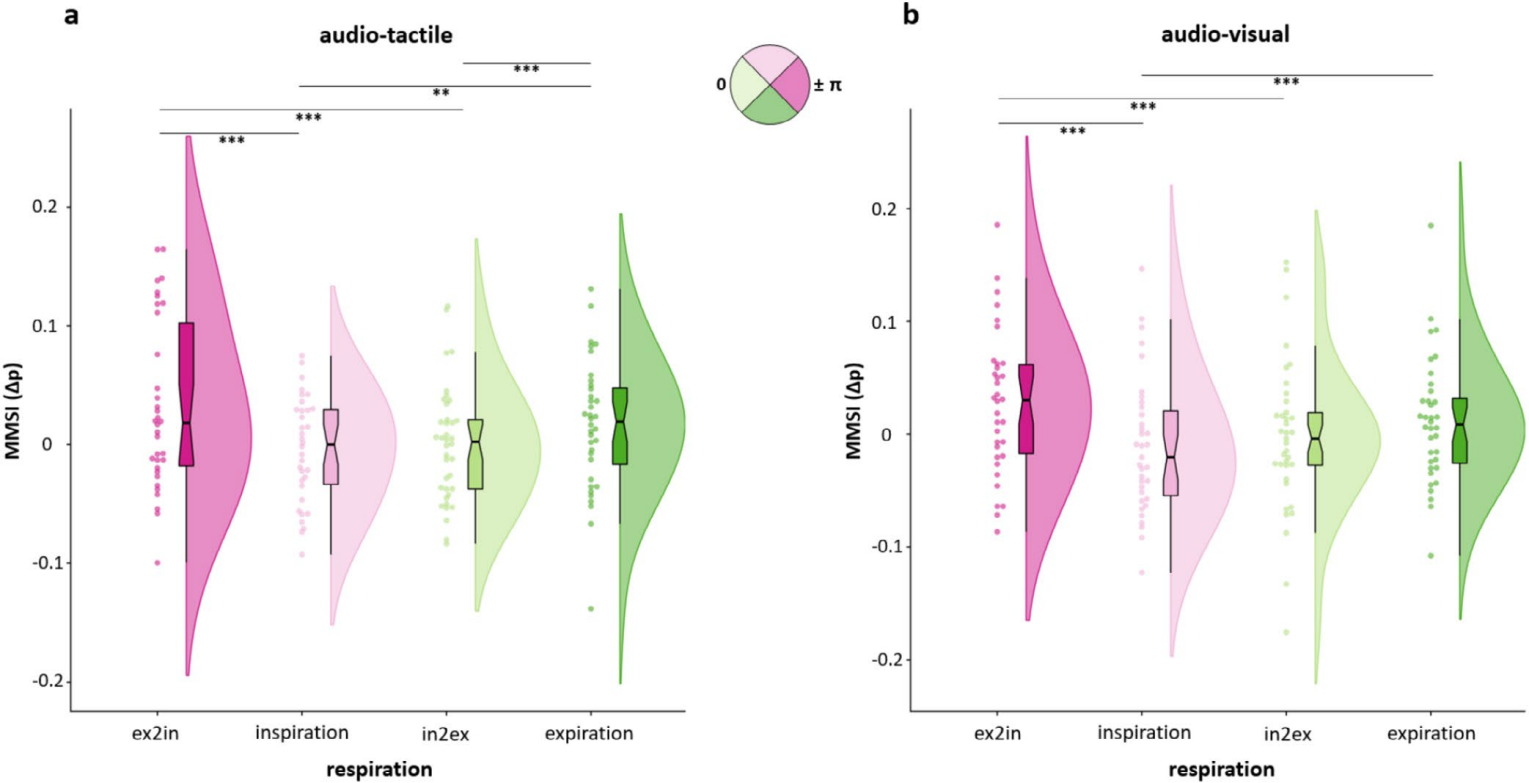
Respiratory modulation of multisensory integration. Both AT (a) and AV (b) magnitude of multisensory integration particularly increased in ex2in and expiration phases, as illustrated by phase‐matched colored raincloud plots. Black lines and asterisks represent significant post hoc comparisons: **p = 0.01, ***p < 0.001.

Regarding AT, the model comparison between the two LMEMs confirmed that including phase information significantly improved the model fit (LR‐stat χ^2^(3) = 38.27, *p* < 0.001) (see Supplementary for the models' stats, Tables S5–S6). The model yielded significant effects of both the AUC (in a time window reaching AUC 11) (*F*(10, 1548) = 4.11, *p* < 0.001) and the respiratory phase (*F*(3, 1548) = 12.92, *p* < 0.001). Respiratory phase clearly influenced AT integration (KW χ^2^(3) = 35.13, *p* < 0.001), with Tukey–Kramer post hoc revealing that ex2in (vs. inspiration mean difference = 137.89, *p* < 0.001; vs. in2ex mean difference = 161.83, *p* < 0.001) and expiration (vs. inspiration mean difference = 101.39, *p* = 0.01; vs. in2ex mean difference = 125.32, *p* < 0.001) exerted the greatest impact (Figure 5a).

In a similar vein, for the AV stimulations, the model including the respiratory phases provided the best fit (LR‐stat χ^2^(3) = 36.50, *p* < 0.001) (see Supplementary for the models' stats, Tables S7–S8). While there was no effect of AUC (up to AUC 10), the respiratory phase significantly modulated multisensory integration as constantly shown by both ANOVA (*F*(3, 1407) = 12.32, *p* < 0.001) and KW test (KW χ^2^(3) = 44.21, *p* < 0.001). Tukey–Kramer post hoc confirmed that this effect was mostly driven by ex2in (vs. inspiration mean difference = 195.26, *p* < 0.001; vs. in2ex mean difference = 138.21, *p* < 0.001) and, to a lesser extent, by expiration (vs. inspiration mean difference = 125.13, *p* < 0.001) (Figure 5b). Overall, these findings indicate a clear role of respiration in shaping multisensory integration, as adding the phase information to the models greatly improved their explanatory power. Moreover, specific respiratory phases seem to boost multisensory integration, suggesting potential interactions with the neural processes underpinning this perceptual feature.

### 3.5 Respiratory Cycle Is Aligned With Response Onset

**FIGURE 6:**
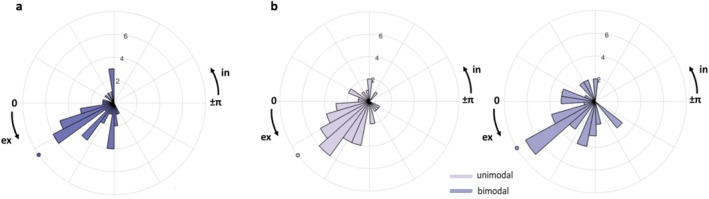
Respiratory cycle is aligned with response onset. (a) Distribution of participants' average phase values at response onset, pooled across stimulus types, shows a preference for early expiration (colored dot: Circular mean). (b) Polar histograms reveal response onset clustering towards early expiration for both unimodal and bimodal stimulation (color‐matched dots: Circular means).

A growing number of empirical findings point to a role of respiration in shaping information sampling and behavior (for recent reviews, see (Brændholt et al. 2023; Heck et al. 2022; Parviainen et al. 2022)). Therefore, we directly tested the association between the respiratory phase and response onsets, as participants may potentially adjust their breathing patterns to meaningful paradigm events. Results showed that, overall, the average phase angles were highly clustered towards expiration and deviated significantly from the null hypothesis of a uniform distribution (*Z*_Rayleigh_ = 17.78, *p* < 0.001, *V*_mean_ = 0.60, 95% CI [0.90 0.30]) (Figure 6a). Inspecting individual data revealed that 24 participants out of 40 systematically aligned their respiratory behavior around response time (the full list is reported in the Table S9). We tested for such an alignment also by splitting data according to the stimulation type (unimodal and bimodal). Circular statistics returned a significant grouping of response onsets average phase angles again within the expiration phase for both unimodal (*Z*_Rayleigh_ = 14.40, *p* < 0.001, *V*_mean_ = 0.64, 95% CI [0.99 0.30]) and bimodal trials (*Z*_Rayleigh_ = 14.14, *p* < 0.001, *V*_mean_ = 0.53, 95% CI [0.88 0.19]) (Figure 6b). These effects were consistent across our study sample, as 17 and 18 participants (out of 40) adapted their respiration to response onset for unimodal and bimodal trials, respectively (*p* < 0.05) (the full list is reported in the Table S9). Taken together, this analysis provides additional evidence of the intricate interplay between respiration and perception. While RTs were found to be higher within the ex2in and lower during inspiration and early expiration, as well as response onsets clustering towards expiration, the ex2in transition would have benefited multisensory integration in both AT and AV conditions.

## 4 Discussion

To address the relationships between respiration and multisensory integration, we tested whether and how respiratory phases relate to various facets of multisensory perception, such as RTs, MMSI, and response onsets to unimodal and bimodal stimuli. We were able to demonstrate that respiration modulates speeded responses and multisensory integration, with participants timing their responses to align with their breathing rhythm.

### 4.1 Distinct Time Windows Within the Respiratory Cycle Speed Up Reaction Times

While research on cardiac interoception has long highlighted the distinct roles of systole and diastole in regulating various cognitive functions (Skora et al. 2022), including sensory (Al et al. 2020; Edwards et al. 2009; Li et al. 2024; Pramme et al. 2016; Ren et al. 2022; Saltafossi et al. 2023; Schulz et al. 2020; Yang et al. 2017) and motor processes (Al et al. 2023; Lai et al. 2024; Marshall et al. 2024; Mussini et al. 2024; Rae et al. 2018), research on respiration is limited. Here, by employing LMEMs and circular statistics, we showed a complex picture of the interplay between the breathing cycle and perception among multiple sensory modalities. Notably, responses were speeded up within two time windows of the cycle, namely around peak inspiration and early expiration. Despite earlier inconclusive outcomes (Flexman et al. 1974; Li et al. 2012), recent investigations on visual information processing have demonstrated that best performances are achieved during inspiration, with some offering neural evidence based on olfactory bulb‐mediated phase‐amplitude coupling to support these effects (Brændholt et al. 2024; Kluger et al. 2021; Perl et al. 2019; Zelano et al. 2016). Various findings have been reported regarding the auditory system. For instance, two studies indicated that the effects of respiration emerged only when participants were required to exert some form of control over their breathing. One study (Gallego et al. 1991) found this resulted in prolonged RTs, while another (Münch et al. 2019) observed faster startle responses to auditory stimuli during expiration. In contrast, somatosensory perception appears to benefit most from later phases of the respiratory cycle. Specifically, the accurate detection of near‐threshold tactile stimuli was enhanced immediately after the onset of expiration (Grund et al. 2022). Despite the variability introduced by differing methods for monitoring respiration (e.g., belts, manometers, or temperature sensors), the specific breathing instructions provided to participants (spontaneous versus controlled), the physical characteristics of the stimuli (near‐threshold versus suprathreshold), and the diverse approaches used to assess respiration‐behavior relationships, our findings align with previous research. Together, they highlight distinct time windows within the respiratory cycle where exteroceptive processing is enhanced.

### 4.2 The Expiration‐To‐Inspiration Transition Slows Down Reaction Times

The other main finding of our study is that speeded responses to both types of stimuli (i.e., unimodal and bimodal) were slowed down during the transition from expiration to inspiration (ex2in). Similarly, a decrease in memory‐related behavioral performance has been pointed out during respiratory phase transitions (Nakamura et al. 2018, 2022). Recall accuracy and RTs to test cues were significantly worsened in a delayed matching‐to‐sample task when the transition from expiration to inspiration occurred during the retrieval process (Nakamura et al. 2018). A neuroimaging study by Nakamura et al. (2022) demonstrated that this ex2in transition during retrieval negatively modulates metabolic responses in critical hubs of the ventral attention network (Corbetta et al. 2008; Marois et al. 2000), including the anterior right temporoparietal junction, right middle frontal gyrus, and dorsomedial prefrontal cortex. In addition to the olfactory bulb‐mediated pathways mentioned earlier, other mechanisms have been proposed to explain how feedback from pontomedullary respiratory centers influences large‐scale brain dynamics during the ex2in phase (Nakamura et al. 2024). For instance, GABAergic inhibition of glutamatergic neurons in the parabrachial nucleus during ex2in downregulates activity in the basal forebrain, hippocampus, and upstream cortical regions (Anaclet et al. 2014, 2015). Moreover, the rhythmic firing of the PreBötzinger Complex (PreBötC), the primary generator of inspiration (Del Negro et al. 2018; Feldman et al. 2013), modulates hippocampal ensemble dynamics and memory performance via abrupt signals evoked during ex2in transitions (Nakamura et al. 2023; Richter and Smith 2014). While previous research has focused on attentional and memory processing, we hypothesize that similar neural pathways underlie the ex2in‐locked increases in RTs observed in our study. Interestingly, both unimodal and bimodal stimuli followed similar respiratory modulation patterns, yet their circular means differed significantly. Functionally, both stimuli were framed within the ex2in phase, although the extent to which respiration uniquely impacts these two sensory processing types remains uncertain.

### 4.3 Multisensory Integration Is Enhanced During the Expiration‐To‐Inspiration Transition

Information coming from multiple senses is combined in our brains to yield multimodally determined percepts (Driver and Spence 2000). This sensory richness provides behavioral advantages, as demonstrated in our study by the Redundancy Signal Effect (RSE). All three crossmodal combinations resulted in faster RTs compared to those observed with single‐modality stimuli. However, when examining the potential super‐additive interactions driving these redundancy gains, only the AT and AV combinations showed evidence of multisensory integration. In contrast, the RMI analysis did not reveal clear evidence of VT integration across different time windows, possibly due to a ceiling effect limiting VT interactions. Multisensory integration typically arises when individual sensory stimuli are weak in eliciting a response on their own, a phenomenon known as ‘inverse effectiveness’ (Meredith and Stein 1986, 1996; Stein and Meredith 1993). Here, the high effectiveness or low variability of V and T stimuli when combined may have diminished the likelihood of VT integration.

LMEMs were applied to multisensory integration (MMSI) indices calculated for the AT and AV combinations. Once again, respiration—categorized into phase transitions (in2ex and ex2in) and non‐transition phases (inspiration and expiration) – was found to predict the MMSI indices, our behavioral proxy for multisensory integration. Unlike RTs facilitation, AT and AV multisensory integration were predominantly enhanced during the ex2in phase, while expiration had a lesser or secondary influence compared to other phases. These findings suggest that respiration plays a role as a physiological modulator of brain activity, potentially influencing neural processes associated with multisensory integration. Specifically, it may act as a “rescue” mechanism during slower responses to bimodal stimuli (Schneeberger et al. 2023). Importantly, this apparent divergence from prior findings that highlighted facilitation during inspiration, particularly for visual perception, may reflect modality‐specific and task‐dependent optimization by respiration. While RTs facilitation was observed during both peak inspiration and expiration in our data (in line with previous unisensory results), multisensory integration appeared to benefit most from the ex2in phase, possibly due to enhanced inhibitory dynamics. Relatedly, it has been recently demonstrated that both oscillatory and non‐oscillatory brain activity covary with the respiratory cycle. For instance, non‐oscillatory fluctuations in cortical excitability, driven by respiratory phases, are marked by changes in the excitation–inhibition (E–I) balance, with steepest slopes, indicative of inhibitory dominance (E < I), around the ex2in transition (Kluger et al. 2023). Multisensory binding also relates to temporal and scale‐free brain activity dynamics (Palva et al. 2013) and to the balance of glutamate and GABA concentrations, which, along with genetic variation in related genes, influence individual variability in AT multisensory integration (Ferri et al. 2017). In auditory–visual paradigms, for example, GABA concentration in the superior temporal sulcus enhances gamma‐band power and AV perceptual binding (Balz et al. 2016). We propose that the ex2in phase fine‐tunes brain activity to align with optimal excitability states (E<I), facilitating multisensory integration.

Our findings speak to recent theoretical proposals like body‐extended multisensory integration, scaffolding, and predictive processing (Engelen et al. 2023), as well as to the multi‐timescale conceptualization of multisensory integration (Senkowski and Engel 2024). In this context, slow rhythms like respiration act as carrier waves, regulating higher cortical dynamics while establishing temporal precision (Allen 2020; Allen et al. 2023; Brændholt et al. 2023; Klimesch 2018). As a consequence, prestimulus functional coupling across both frequency bands and brain regions (Galindo‐Leon et al. 2019; Keil et al. 2012, 2014; Leonardelli et al. 2015), and fluctuations in ongoing oscillations that support multisensory integration (Buergers and Noppeney 2022; Cecere et al. 2015; Keil and Senkowski 2017) may be intrinsically orchestrated by respiration. This coordination might impose specific brain states within which multisensory processing occurs.

### 4.4 Response Onsets Cluster Within Early Expiration

Building on concepts like embodied predictive interoception coding (EPIC) (Barrett and Simmons 2015) and interoceptive active inference (Ainley et al. 2016; Allen et al. 2023) which constrain model updating and PEs minimization to visceral ascending and descending information, our results suggest that response timing is intricately linked to specific respiratory phases, particularly during early expiration. Given respiration's predictability and adaptability (Goheen et al. 2024), our findings highlight how sensory sampling and environmental interactions align with specific brain–body states (Criscuolo et al. 2022; Galvez‐Pol et al. 2020, 2022; Kluger et al. 2021). This matches the active sensing hypothesis and experimental results showing that task, stimulus, response onsets, and actions triggered externally or through mental imagery synchronize with respiration (Grund et al. 2022; Harting et al. 2024; Johannknecht and Kayser 2022; Park et al. 2020, 2022; Perl et al. 2019; Stetza et al. 2024; Zelano et al. 2016).

While we cannot definitively establish directionality between response clustering and RTs reduction, these phenomena likely reflect respiration‐based precision‐weighting mechanisms. In predictive processing, precision reflects confidence in predictions and prediction errors, modulated via neurotransmitter activity (Ferguson and Cardin 2020; Moran et al. 2013; Pinotsis et al. 2019; Warren et al. 2016). Notably, brainstem breathing circuits directly elicit a noradrenergic release from the locus coeruleus, tightly coupling respiration to arousal and behavioral regulation (Kluger, Gross, and Keitel 2024; Melnychuk et al. 2018; Schaefer et al. 2023, 2024; Yackle et al. 2017). This may explain the cognitive and attentional benefits observed with controlled breathing practices in traditions like yoga and martial arts, where respiratory regulation enhances focus and clarity (Brown and Gerbarg 2005; Li and Laskin 2006; Varga and Heck 2017; Zaccaro et al. 2018).

## 5 Conclusion

In conclusion, our findings strongly suggest that respiration actively shapes exteroceptive processing, encompassing both unisensory and multisensory inputs. Future research should aim to disentangle the respiratory entrainment of responses from genuine multisensory integration, while also identifying the specific neurophysiological mechanisms supporting both processes. This would help substantiate a more comprehensive perspective on multisensory integration extending to an embodied hierarchical neuroarchitecture in which slower bodily rhythms play a crucial role (Banellis et al. 2024; Candia‐Rivera et al. 2024; Kluger, Allen, and Gross 2024; Rebollo and Tallon‐Baudry 2022; Rebollo et al. 2018). Such insights could also advance our understanding of neurological and psychiatric disorders characterized by disrupted interoceptive processing, where dysfunctional brain–body coupling can hinder adaptive behavior (Khalsa et al. 2018; Kluger et al. 2025; Nord and Garfinkel 2022; Paulus et al. 2019; Yao and Thakkar 2022). In this context, potential applications include incorporating breathing exercises into treatment programs, enhancing engagement in anxiety and PTSD interventions through respiratory‐aligned stimulus delivery, and using biofeedback to synchronize cognitive or motor training with the respiratory cycle (Saltafossi et al. 2025; Schoeller et al. 2024). In this context, potential applications include incorporating breathing exercises into treatment programs, enhancing engagement in anxiety and PTSD (post‐traumatic stress disorder) interventions through respiratory‐aligned stimulus delivery, and using biofeedback to synchronize cognitive or motor training with the respiratory cycle (Saltafossi et al. 2025; Schoeller et al. 2024).

## Author Contributions

**Martina Saltafossi:** data curation, formal analysis, investigation, methodology, validation, visualization, writing – original draft, writing – review and editing. **Andrea Zaccaro:** conceptualization, methodology, writing – review and editing. **Daniel S. Kluger:** methodology, visualization, writing – review and editing. **Mauro Gianni Perrucci:** resources, software, writing – review and editing. **Francesca Ferri:** conceptualization, funding acquisition, supervision, writing – review and editing. **Marcello Costantini:** conceptualization, funding acquisition, project administration, supervision, writing – review and editing.

## Conflicts of Interest

The authors declare no conflicts of interest.

## Supporting information


**Data S1:** psyp70145‐sup‐0001‐DataS1.docx.

## Data Availability

The data that support the findings of this study are available from the corresponding author upon reasonable request.
