# Supplementary material for: Respiration Facilitates Behavior During Multisensory Integration
Source: Psychophysiology. 2025 Sep 25;62(9):e70145. doi: 10.1111/psyp.70145 (PMC12464358; doi:10.1111/psyp.70145)
Supplement: Supplementary file 1 — Data S1: psyp70145‐sup‐0001‐DataS1.docx. [file PSYP-62-e70145-s001.docx]

**Supplementary**

# 1. Experimental setup - stimuli and thresholding procedure description

The stimulus A was a pure tone (1000 Hz; 30 msec of duration) presented approximately at 60 dB using a buzzer; V stimulus (30 msec of duration) was delivered through a light-emitting diode (LED) (5 mm diameter; 200 mcd); T stimulus consisted of a suprathreshold electrical pulse (100 µs of duration), delivered on the middle finger of the right hand using a Digitimer (DS7A, Digitimer Ltd., Welwyn Garden City, UK). A and V stimuli were presented through an in-house box containing a fixation cross, the buzzer, and the LED (main text: **Figure 1a**). Stimuli presentation was controlled with the E-Prime 3.0 software (Psychology Software Tools, Pittsburgh, PA, USA) connected to a TriggerStation™ (BRAINTRENDS LTD 2010, Rome, Italy). Individual thresholds of the tactile stimulus were set to be clearly suprathreshold using the method of the limits [(Gerr & Letz, 1988)](https://sciwheel.com/work/citation?ids=15359940&pre=&suf=&sa=0). Before starting the experiment, the intensity of the stimulator was set to 0 mA and then progressively increased by 1 mA until the subject reported to clearly perceive the stimulation. Then, the participant was additionally stimulated 5 times: as soon as one of the additional stimuli was not detected, the intensity was increased by 1 mA, and the procedure was repeated [(Saltafossi et al., 2023; Vastano et al., 2022)](https://sciwheel.com/work/citation?ids=15359945,15445821&pre=&pre=&suf=&suf=&sa=0,0).

# Friedman’s tests for Redundant Signals Effect

## Table 1. F-tests and post hoc (Tukey Kramer)

| **modality triplets** | **F-test stats** | | | | | |
| --- | --- | --- | --- | --- | --- | --- |
| **A/T/AT** | **source** | **SS** | **DF** | **MS** | **Chi-sq** | **p-value** |
|  | columns | 72.8 | 2 | 36.4 | 72.8 | < .001 |
|  | error | 7.2 | 78 | 0.09 |  |  |
|  | total | 80 | 119 |  |  |  |
|  | **post hoc (Tukey Kramer)** | | | | | |
|  | **stim type** | **stim type** | **low limit** | **difference** | **upper limit** | **p-value** |
|  | T | A | 0.27 | 0.80 | 1.32 | < .001 |
|  | T | AT | 1.37 | 1.90 | 2.42 | < .001 |
|  | A | AT | 0.57 | 1.10 | 1.62 | < .001 |
|  | **F-test stats** | | | | | |
| **A/V/AV** | **source** | **SS** | **DF** | **MS** | **Chi-sq** | **p-value** |
|  | columns | 60.8 | 2 | 30.4 | 60.8 | < .001 |
|  | error | 19.2 | 78 | 0.25 |  |  |
|  | total | 80 | 119 |  |  |  |
|  | **post hoc (Tukey Kramer)** | | | | | |
|  | **stim type** | **stim type** | **low limit** | **difference** | **upper limit** | **p-value** |
|  | V | A | -0.32 | 0.20 | 0.72 | .643 |
|  | V | AV | 1.07 | 1.60 | 2.12 | < .001 |
|  | A | AV | 0.87 | 1.40 | 1.92 | < .001 |
|  | **F-test stats** | | | | | |
| **V/T/VT** | **source** | **SS** | **DF** | **MS** | **Chi-sq** | **p-value** |
|  | columns | 61.85 | 2 | 30.92 | 61.85 | < .001 |
|  | error | 18.15 | 78 | 0.24 |  |  |
|  | total | 80 | 119 |  |  |  |
|  | **post hoc (Tukey Kramer)** | | | | | |
|  | **stim type** | **stim type** | **low limit** | **difference** | **upper limit** | **p-value** |
|  | T | V | 0.50 | 1.02 | 1.54 | < .001 |
|  | T | VT | 1.22 | 1.75 | 2.27 | < .001 |
|  | V | VT | 0.20 | 0.72 | 1.24 | .003 |

##

# Linear Mixed Effect Models (LMEMs) for RTs and respiration

## Table 2. fixed effects coefficients from alternative-full model = clean RTs ~ 1 + stimulus type + sine + cosine + (1|participant)

| **name** | **estimate** | **SE** | **tStat** | **DF** | **p-value** | **lower** | **upper** |
| --- | --- | --- | --- | --- | --- | --- | --- |
| intercept | 410.61 | 11.80 | 34.78 | 4796 | < .001 | 387.47 | 433.76 |
| stimulus type | -48.63 | 0.36 | -135.74 | 4796 | < .001 | -49.34 | -47.93 |
| cosine | -2.85 | 0.25 | -11.34 | 4796 | < .001 | -3.34 | -2.36 |
| sine | .65 | 0.26 | 2.53 | 4796 | .012 | 0.14 | 1.15 |

## Table 3. Model comparison: Theoretical Likelihood Ratio Test

| **model** | **DF** | **AIC** | **BIC** | **Log-Likelihood** | **LR-Stat** | **delta df** | **p-value** |
| --- | --- | --- | --- | --- | --- | --- | --- |
| base | 4 | 38277 | 38303 | -19135 |  |  |  |
| full | 6 | 38148 | 38187 | -19068 | 133.16 | 2 | < .001 |

## Figure S1. Alternative-full model empirical vs null distributions

Histogram shows the empirical LMEM beta for the respiratory vector norm against the null distribution computed from 1000 iterations of randomized RTs vectors on the subject level.


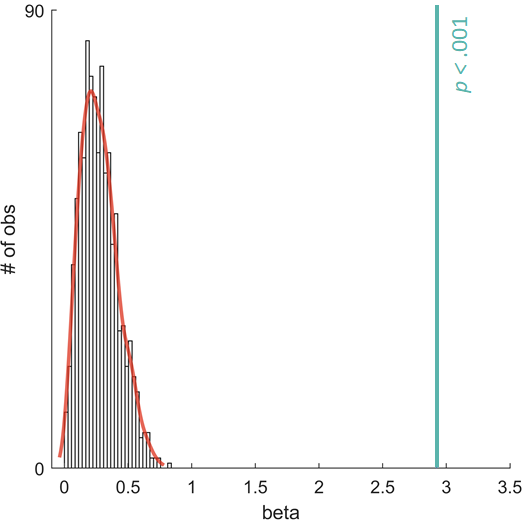


# Circular Analyses (RTs)

## Table 4. Bins t-stats with FDR corrected p-values

The following table lists overall, unimodal, and bimodal 2-tailed t-stat values, one for each phase bin (from 1 to 60), with corresponding FDR-adjusted p-values. The yellow filling represents significant (i.e. *p*_FDR_  < .05) t-stat > 0 (slow RTs) while the green filling represents significant (i.e. *p*_FDR_  < .05) t-stat < 0 (fast RTs).

|  | **overall** | **unimodal** | | **bimodal** | |  |  |
| --- | --- | --- | --- | --- | --- | --- | --- |
| **t-stat** | **p-value (FDR)** | **t-stat** | **p-value (FDR)** | **t-stat** | **p-value (FDR)** | **rad** | **bin** |
| 5.14 | < .001 | 2.79 | .036 | 3.38 | .008 | -3.14 | 1 |
| 3.90 | .001 | 2.54 | .051 | 2.95 | .006 | -3.04 | 2 |
| 3.54 | .001 | 2.26 | .052 | 1.63 | .044 | -2.93 | 3 |
| 2.99 | .004 | 2.81 | .041 | 0.16 | .147 | -2.82 | 4 |
| 1.70 | .032 | 2.20 | .049 | -0.22 | 0.141 | -2.72 | 5 |
| 1.37 | .050 | 1.92 | .067 | -0.28 | 0.135 | -2.61 | 6 |
| 1.35 | .051 | 2.01 | .066 | -0.33 | .131 | -2.50 | 7 |
| 0.60 | .118 | 1.56 | .112 | -0.55 | 0.112 | -2.40 | 8 |
| -0.38 | .138 | 0.90 | .239 | -1.10 | 0.074 | -2.29 | 9 |
| -1.48 | .045 | 0.40 | .362 | -1.86 | .031 | -2.18 | 10 |
| -1.93 | .022 | -1.16 | .182 | -1.44 | .048 | -2.08 | 11 |
| -1.98 | .021 | -0.54 | .328 | -1.99 | .026 | -1.97 | 12 |
| -2.05 | .020 | -0.31 | .379 | -2.26 | .02 | -1.86 | 13 |
| -2.88 | .005 | -2.19 | .048 | -2.31 | .019 | -1.76 | 14 |
| -2.82 | .005 | -1.98 | .061 | -1.92 | .029 | -1.65 | 15 |
| -2.77 | .005 | -2.51 | .049 | -1.50 | .047 | -1.54 | 16 |
| -1.37 | .051 | -1.39 | .144 | -0.70 | .109 | -1.44 | 17 |
| -1.98 | .021 | -1.72 | .085 | -0.67 | .11 | -1.33 | 18 |
| -1.18 | .064 | -1.16 | .177 | -1.07 | .075 | -1.22 | 19 |
| -1.16 | .064 | -1.51 | .120 | -0.52 | .114 | -1.12 | 20 |
| -1.28 | .055 | -1.36 | .146 | -0.74 | .109 | -1.01 | 21 |
| -0.63 | .119 | -0.94 | .235 | -0.56 | .116 | -0.91 | 22 |
| 0.07 | .175 | 0.17 | .420 | -0.89 | .091 | -0.80 | 23 |
| 0.02 | .179 | -0.22 | .409 | -0.34 | .132 | -0.69 | 24 |
| -0.13 | .170 | -0.35 | .371 | -0.03 | .153 | -0.59 | 25 |
| 0.60 | .121 | 0.86 | .245 | -0.67 | .109 | -0.48 | 26 |
| 0.36 | .138 | 0.49 | .341 | 0.40 | .127 | -0.37 | 27 |
| -0.53 | .122 | -0.05 | .433 | 0.11 | .148 | -0.27 | 28 |
| -0.78 | .100 | -0.10 | .421 | -0.64 | .109 | -0.16 | 29 |
| -1.01 | .077 | 0.17 | .413 | -1.34 | .055 | -0.05 | 30 |
| -1.49 | .045 | -0.74 | .269 | -0.56 | .114 | 0.05 | 31 |
| -2.08 | .019 | -0.59 | .316 | -1.22 | .065 | 0.16 | 32 |
| -1.64 | .035 | -1.11 | .187 | -0.89 | .093 | 0.27 | 33 |
| -1.74 | .032 | -1.29 | .159 | -0.96 | .087 | 0.37 | 34 |
| -2.03 | .020 | -1.74 | .088 | -1.20 | .065 | 0.48 | 35 |
| -3.14 | .003 | -2.45 | .051 | -2.51 | .014 | 0.59 | 36 |
| -3.28 | .002 | -2.32 | .052 | -2.36 | .018 | 0.69 | 37 |
| -2.66 | .006 | -2.32 | .049 | -1.76 | .036 | 0.80 | 38 |
| -2.67 | .006 | -2.21 | .054 | -2.12 | .021 | 0.91 | 39 |
| -2.57 | .007 | -1.98 | .064 | -2.21 | .021 | 1.01 | 40 |
| -2.46 | .009 | -2.00 | .063 | -2.17 | .02 | 1.12 | 41 |
| -2.42 | .010 | -2.35 | .053 | -1.47 | .049 | 1.22 | 42 |
| -2.71 | .006 | -2.40 | .051 | -1.59 | .043 | 1.33 | 43 |
| -1.73 | .031 | -2.20 | .053 | -0.60 | .113 | 1.44 | 44 |
| -1.09 | .071 | -1.23 | .171 | -0.10 | .147 | 1.54 | 45 |
| -0.83 | .096 | -0.75 | .271 | 0.15 | .145 | 1.65 | 46 |
| -0.56 | .119 | -0.90 | .243 | 0.72 | .109 | 1.76 | 47 |
| 0.43 | .133 | -0.42 | .359 | 1.60 | .044 | 1.86 | 48 |
| 0.88 | .091 | 0.01 | .442 | 1.58 | .042 | 1.97 | 48 |
| 0.59 | .117 | -0.11 | .428 | 1.46 | .048 | 2.08 | 50 |
| 1.40 | .050 | 0.85 | .242 | 1.77 | .036 | 2.18 | 51 |
| 2.23 | .014 | 1.22 | .171 | 2.60 | .014 | 2.29 | 52 |
| 3.28 | .002 | 1.84 | .075 | 3.19 | .007 | 2.40 | 53 |
| 3.46 | .002 | 1.73 | .087 | 3.40 | .015 | 2.50 | 54 |
| 3.43 | .002 | 2.10 | .056 | 2.59 | .013 | 2.61 | 55 |
| 4.42 | < .001 | 2.92 | .038 | 2.19 | .02 | 2.72 | 56 |
| 5.18 | < .001 | 3.13 | .029 | 3.12 | .005 | 2.82 | 57 |
| 5.89 | < .001 | 3.73 | .016 | 3.14 | .005 | 2.93 | 58 |
| 5.57 | < .001 | 3.44 | .019 | 3.16 | .006 | 3.04 | 59 |
| 5.14 | < .001 | 2.79 | .031 | 3.38 | .005 | 3.14 | 60 |

# Linear Mixed Effect Models (LMEMs) for MMSI (magnitude of multisensory integration) and respiration

## Audio-tactile MMSI

### Table 5. ANOVA marginal tests (on coefficients) from alternative-full model = MMSI ~ 1 + phase + AUC + (1|participant)

| **term** | **F-stat** | **DF1** | **DF2** | **p-value** |
| --- | --- | --- | --- | --- |
| intercept | 1.10 | 1 | 1548 | .295 |
| AUC | 4.11 | 10 | 1548 | < .001 |
| phase | 12.92 | 3 | 1548 | < .001 |

### Table 6. Model comparison: Theoretical Likelihood Ratio Test

| **model** | **DF** | **AIC** | **BIC** | **Log-Likelihood** | **LR-Stat** | **delta df** | **p-value** |
| --- | --- | --- | --- | --- | --- | --- | --- |
| base | 13 | -3455.6 | -3386 | 1740.8 |  |  |  |
| full | 16 | -3487.9 | -3402.2 | 1759.9 | 38.267 | 3 | < .001 |

## Audio-visual MMSI

### Table 7. ANOVA marginal tests (on coefficients) from alternative-full model = MMSI ~ 1 + phase + AUC + (1|participant)

| **term** | **F-stat** | **DF1** | **DF2** | **p-value** |
| --- | --- | --- | --- | --- |
| intercept | 2.44 | 1 | 1407 | .118 |
| AUC | 0.88 | 9 | 1407 | .540 |
| phase | 12.32 | 3 | 1407 | < .001 |

### Table 8. Model comparison: Theoretical Likelihood Ratio Test

| **model** | **DF** | **AIC** | **BIC** | **Log-Likelihood** | **LR-Stat** | **delta df** | **p-value** |
| --- | --- | --- | --- | --- | --- | --- | --- |
| base | 12 | -2991.3 | -2928.2 | 1507.6 |  |  |  |
| full | 15 | -3021.8 | -2942.9 | 1525.9 | 36.503 | 3 | < .001 |

# Circular Analyses (Response onset)

## Table 9. Consistency across participants (Response onset)

The following table lists overall, unimodal, and bimodal Z-stats from Rayleigh tests, one for each participant (from 1 to 40), with corresponding p-values. The red filling represents significant (i.e. *p* < .05) non-uniform distribution of the data (response onsets).

| **Rayleigh Z** | **p-value** | **unimodal** | | **bimodal** | |
| --- | --- | --- | --- | --- | --- |
| 11.05 | < .001 | **Rayleigh Z** | **p-value** | **Rayleigh Z** | **p-value** |
| 1.54 | .215 | 6.48 | .001 | 5.06 | .006 |
| 10.22 | < .001 | 2.89 | .056 | 0.03 | .974 |
| 0.69 | .502 | 5.37 | .005 | 4.96 | .007 |
| 2.57 | .076 | 1.21 | .297 | 0.05 | .954 |
| 0.62 | .541 | 1.15 | .316 | 1.64 | .193 |
| 16.62 | < .001 | 0.32 | .727 | 0.30 | .742 |
| 2.08 | .125 | 8.97 | < .001 | 7.84 | < .001 |
| 3.83 | .022 | 0.88 | .417 | 2.60 | .075 |
| 0.40 | .671 | 3.33 | .036 | 0.97 | .381 |
| 1.65 | .192 | 0.13 | .875 | 1.21 | .299 |
| 6.57 | .001 | 1.71 | .181 | 0.28 | .758 |
| 2.82 | .059 | 2.57 | .076 | 4.16 | .015 |
| 1.13 | .324 | 2.83 | .059 | 0.63 | .535 |
| 16.69 | < .001 | 1.12 | .326 | 0.86 | .425 |
| 2.07 | .126 | 7.70 | < .001 | 9.95 | < .001 |
| 11.86 | < .001 | 1.40 | .246 | 1.60 | .203 |
| 3.35 | .035 | 7.98 | < .001 | 4.19 | .015 |
| 19.20 | < .001 | 1.67 | .188 | 1.90 | .150 |
| 3.41 | .033 | 12.53 | < .001 | 7.06 | .001 |
| 2.12 | .119 | 1.51 | .221 | 3.84 | .021 |
| 10.21 | < .001 | 2.32 | .098 | 0.57 | .567 |
| 10.14 | < .001 | 4.74 | .009 | 5.50 | .004 |
| 11.17 | < .001 | 6.95 | .001 | 3.51 | .030 |
| 6.30 | .002 | 4.47 | .011 | 6.89 | .001 |
| 2.09 | .124 | 0.10 | .907 | 10.32 | < .001 |
| 0.73 | .480 | 2.93 | .053 | 2.37 | .093 |
| 12.32 | < .001 | 0.65 | .522 | 0.74 | .479 |
| 8.76 | < .001 | 3.65 | .026 | 9.68 | < .001 |
| 4.36 | .013 | 2.93 | .053 | 6.09 | .002 |
| 0.53 | .588 | 4.39 | .012 | 0.93 | .394 |
| 69.42 | < .001 | 0.22 | .802 | 0.32 | .727 |
| 2.37 | .093 | 41.29 | < .001 | 30.64 | < .001 |
| 4.07 | .017 | 1.22 | .296 | 1.47 | .229 |
| 5.58 | .004 | 0.64 | .529 | 4.22 | .015 |
| 0.57 | .565 | 2.31 | .099 | 3.56 | .028 |
| 13.04 | < .001 | 1.10 | .334 | 0.00 | .995 |
| 10.15 | < .001 | 8.73 | < .001 | 4.71 | .009 |
| 12.49 | < .001 | 9.10 | < .001 | 2.40 | .090 |
| 6.27 | .002 | 11.37 | < .001 | 2.79 | .061 |
|  |  | 6.11 | .002 | 1.49 | .225 |

# References

[Gerr, F. E., & Letz, R. (1988). Reliability of a widely used test of peripheral cutaneous vibration sensitivity and a comparison of two testing protocols. *British Journal of Industrial Medicine*, *45*(9), 635–639. https://doi.org/10.1136/oem.45.9.635](https://sciwheel.com/work/bibliography/15359940)

[Saltafossi, M., Zaccaro, A., Perrucci, M. G., Ferri, F., & Costantini, M. (2023). The impact of cardiac phases on multisensory integration. *Biological Psychology*, *182*, 108642. https://doi.org/10.1016/j.biopsycho.2023.108642](https://sciwheel.com/work/bibliography/15445821)

[Vastano, R., Costantini, M., Alexander, W. H., & Widerstrom-Noga, E. (2022). Multisensory integration in humans with spinal cord injury. *Scientific Reports*, *12*(1), 22156. https://doi.org/10.1038/s41598-022-26678-x](https://sciwheel.com/work/bibliography/15359945)
